# Mechanisms of Thymoquinone Hepatorenal Protection in Methotrexate-Induced Toxicity in Rats

**DOI:** 10.1155/2015/859383

**Published:** 2015-05-21

**Authors:** Azza A. K. El-Sheikh, Mohamed A. Morsy, Ahlam M. Abdalla, Azza H. Hamouda, Ibrahim A. Alhaider

**Affiliations:** ^1^Department of Pharmacology, Faculty of Medicine, Minia University, El-Minia 61511, Egypt; ^2^Department of Pharmaceutical Sciences, College of Clinical Pharmacy, King Faisal University, Al-Ahsa 31982, Saudi Arabia; ^3^Department of Biochemistry, Faculty of Medicine, Minia University, El-Minia 61511, Egypt; ^4^Department of Histology, Faculty of Medicine, Minia University, El-Minia 61511, Egypt

## Abstract

To investigate mechanisms by which thymoquinone (TQ) can prevent methotrexate- (MTX-) induced hepatorenal toxicity, TQ (10 mg/kg) was administered orally for 10 days. In independent rat groups, MTX hepatorenal toxicity was induced via 20 mg/kg i.p. at the end of day 3 of experiment, with or without TQ. MTX caused deterioration in kidney and liver function, namely, blood urea nitrogen, creatinine, alanine aminotransferase, and aspartate aminotransferase. MTX also caused distortion in renal and hepatic histology, with significant oxidative stress, manifested by decrease in reduced glutathione and catalase, as well as increase in malondialdehyde levels. In addition, MTX caused nitrosative stress manifested by increased nitric oxide, with upregulation of inducible nitric oxide synthase. Furthermore, MTX caused hepatorenal inflammatory effects as shown by increased tumor necrosis factor-*α*, besides upregulation of necrosis factor-*κ*B and cyclooxygenase-2 expressions. MTX also caused apoptotic effect, as it upregulated caspase 3 in liver and kidney. Using TQ concurrently with MTX restored kidney and liver functions, as well as their normal histology. TQ also reversed oxidative and nitrosative stress, as well as inflammatory and apoptotic signs caused by MTX alone. Thus, TQ may be beneficial adjuvant that confers hepatorenal protection to MTX toxicity via antioxidant, antinitrosative, anti-inflammatory, and antiapoptotic mechanisms.

## 1. Introduction

Methotrexate (MTX) is one of the most widely used anticancer drugs. Unfortunately, cytotoxic effects of MTX may not only affect tumor cells, but also extend to affect vital organs. One of the most prominent toxicities caused by MTX chemotherapy is nephrotoxicity [1, 2]. MTX-induced renal dysfunction is initiated by the precipitation of MTX and its metabolites in the renal tubules. Consequently, MTX concentration is elevated in plasma, as MTX depends mainly on active renal tubular transport for its excretion [3]. The elevated toxic levels of MTX then contribute to multiple vital organ damage, including the liver. Sadly, the prevalence of MTX-induced liver fibrosis and cirrhosis in patients may reach up to 50% and 26%, respectively [4]. Finding an adjuvant hepatorenal protective compound is, thus, mandatory for the safe use of this indispensable anticancer immunosuppressant drug.

The black seed (*Nigella sativa*), a nutritional flavoring agent, is known to have healing potentials and has been used as folk medicine in middle and far east for a wide range of diseases [5]. Thymoquinone (TQ), 2-isopropyl-5-methyl-1,4-benzoquinone, is one of the active ingredients of* Nigella sativa* seeds. Several studies were performed on* Nigella sativa* extract and TQ that suggest that their pharmacological effects include anticancer activity [6]. These findings suggest TQ as an adjuvant compound to cancer chemotherapy.

Here, we propose to investigate the effect of TQ on hepatorenal toxic damage induced by MTX. In addition, we explore the possible mechanisms involved, as the role of oxidative and nitrosative stress, as well as the contribution of inflammatory mediators as tumor necrosis factor- (TNF-) *α*, nuclear factor- (NF-) *κ*B and cyclooxygenase- (COX-) 2, and the apoptotic marker; caspase 3.

## 2. Materials and Methods

### 2.1. Chemicals

TQ was purchased from Sigma-Aldrich Corp. (MO, USA), MTX by Minapharm Pharmaceuticals (Egypt), and TNF-*α* enzyme-linked immunosorbent assay (ELISA) kit by Wkea Med Supplies Corp. (China). Kits for examining blood urea nitrogen (BUN), creatinine, alanine aminotransferase (ALT), aspartate aminotransferase (AST), reduced glutathione (GSH), and catalase were purchased from Biodiagnostic (Egypt). The ready to use inducible nitric oxide synthase (iNOS), NF-*κ*B/p65, COX-2, and caspase 3 rabbit polyclonal antibodies were purchased from Thermo Fisher Scientific Inc./Lab Vision (CA, USA).

### 2.2. Experimental Design

Thirty-eight adult male Wistar rats of 190–240 g weight were purchased from the National Research Center (Giza, Egypt). All animal care and experimental procedures were in accordance with the protocols of the Research Advisory Ethical Committee of Faculty of Medicine, Minia University, Egypt, and EU Directive 2010/63/EU. Rats were housed as 3 or 4 rats/cage in the standard animal facility during the whole experiment, where they had free access to commercial laboratory chow and tap water. Animals were left to acclimatize for 2 weeks before the start of experiment. Afterwards, animals were weighed and divided into 4 groups. TQ-treated group (*n* = 8) received a single daily oral dose of 10 mg/kg/day TQ by gastric gavage for 10 consecutive days [7]. MTX-treated group (*n* = 11) received a single i.p. dose of 20 mg/kg MTX at the end of day 3 of the experiment [8]. Combined MTX/TQ-treated group (*n* = 11) received both MTX and TQ treatments as previously indicated. Untreated group (*n* = 8) served as control. Vehicles for both TQ and MTX were given to control and respective groups not receiving either drug.

### 2.3. Sample Preparation

After 7 days of MTX injection, total rat body weights were recorded at the end of the experiment. Rats were sacrificed by cervical dislocation and blood samples were collected and centrifuged at 5000 rpm for 15 min. Serum was then collected and stored at −80°C until use. Both kidneys and liver were rapidly excised and weighed. Kidney and liver sections were fixed in 10% formalin and embedded in paraffin for histopathological and immunohistochemical examinations. The rest of the kidney and liver tissues were snap-frozen in liquid nitrogen and kept at −80°C. To prepare tissue homogenate, the liver and kidneys were homogenized using Glas-Col homogenizer and a 20% w/v homogenate was prepared in ice-cold phosphate buffer (0.01 M, pH 7.4). The homogenate was centrifuged at 3000 rpm for 20 min and the supernatant was aliquoted to avoid sample thawing and refreezing and was kept at −80°C until use.

### 2.4. Evaluation of Serum Kidney/Liver Function and Tissue Oxidative Stress Markers

Renal function and nephrotoxicity were assessed via determination of BUN and serum creatinine, whereas liver function and hepatotoxicity were evaluated by serum ALT and AST using colorimetric diagnostic kits according to the manufacturer's instructions. Biochemical oxidative stress markers were determined in tissue homogenate of kidney and liver, where GSH concentration, catalase activity, and lipid peroxide content were evaluated. A spectrophotometric kit was used for assessment of GSH. Briefly, the method is based on the fact that the sulfhydryl component of GSH reacts with 5,5-dithio-bis-2-nitrobenzoic acid (Ellman's reagent) producing 5-thio-2-nitrobenzoic acid having a yellow color that was measured colorimetrically at 405 nm (Beckman DU-64 UV/VIS spectrophotometer). Results were expressed as *μ*mol/g tissue. Assessment of catalase antioxidant enzymatic activity was determined in tissue homogenate from the rate of decomposition of H_2_O_2_ at 510 nm. The results were expressed as unit/g tissue. Tissue content of lipid peroxides was determined by biochemical assessment of thiobarbituric acid reacting substance through spectrophotometric measurement of color at 535 nm, using 1,1,3,3-tetramethoxypropane as standard [9]. The results were expressed as equivalents of malondialdehyde (MDA) in tissue homogenate in nmol/g tissue.

### 2.5. Assessment of Nitrosative Stress Marker and TNF-*α* in Tissue Homogenate

For the assessment of nitrosative stress in rat kidney and liver homogenate, the stable oxidation end products of nitric oxide (NO), nitrite, and nitrate were used as an index of NO production, as NO has a half-life of only a few seconds, being readily oxidized to nitrite and then to nitrate. The method used was based on Griess reaction [10] which depends on conversion of nitrate into nitrite by copperized cadmium granules and then measuring the total nitrites spectrophotometrically at 540 nm. Results were expressed as nmol/100 mg tissue. TNF-*α* was determined according to ELISA kit manufacturer's instructions. TNF-*α* was assessed in 10 *μ*L of kidney or liver homogenate using the supplied 96-well ELISA plate. The plate was read using ELISA plate reader at 450 nm.

### 2.6. Histopathological and Immunohistochemical Examination

The specimens from the kidney and liver were collected and fixed in 10% formalin solution and embedded in paraffin. Five *μ*m thick paraffin sections were prepared and then routinely stained with hematoxylin and eosin (H&E) dyes. Stained slides were microscopically analyzed using light microscopy (Olympus CX41). For immunohistochemical staining, sections were fixed at 65°C for 1 h. Triology pretreatment (deparaffinization, rehydration, and antigen unmasking) was used to enhance standardization of the pretreatment step and produce results that are more consistent. The rabbit polyclonal antibodies against iNOS, NF-*κ*B, COX-2, and caspase 3 were employed as it is (ready to use) according to their manufacturer's specification. After applying the antibodies, slides were incubated overnight at 4°C followed by 20 min of Poly HRP enzyme conjugation. Afterwards, DAB chromogen was applied for 2 min and then rinsed, followed by counterstaining with Mayer hematoxylin before examination under the light microscope. Using ImageJ 1.41 (freeware; rsbweb.nih.gov/ij), the total number of cells in a field was calculated by counting hematoxylin-positive cells using ImageJ particle count command. The immunopositive cells were also counted the same way after performing color deconvolution command. Results were the average of counting three sections from each rat, with five rats per condition, and were expressed as percent of immunopositive cells compared to total number of cells.

### 2.7. Statistical Analysis

The data were analyzed by one way ANOVA followed by Dunnett Multiple Comparison Test. The values are represented as means ± SEM. All statistical analyses were done using GraphPad Prism software, version 5.00 (San Diego, CA, USA). The differences were considered significant when the calculated *p* value is less than 0.05.

## 3. Results

### 3.1. Effect of TQ on Change in Total Body Weight, Organ/Body Weight Ratio, and Hepatorenal Function in MTX-Induced Toxicity

Total body weight was measured at the beginning and at the end of the experiment. The percent of change of body weight between the initial and final weights was significantly lower in MTX-treated group compared to control (Table 1). Administration of TQ with MTX significantly improved the percent of change of total body weight compared to MTX sole therapy. The ratio between the weight of the kidney and the final body weight was significantly higher in MTX group compared to control, which was significantly reversed in combined MTX/TQ-treated group. Similarly, MTX caused significant increase in liver/total body weight ratio compared to control, which was reversed by combined pretreatment with TQ. Interestingly, TQ pretreatment improved the kidney/weight ratio more than that of the liver, as the former decreased to levels not significantly different from control. Renal function markers, BUN and creatinine, and of liver function makers, ALT and AST, were significantly elevated in MTX-treated group compared to control. Combined MTX/TQ treatment significantly improved renal and hepatic function markers compared to sole MTX treatment. It was noticed that the improvement of the kidney function was more pronounced than that of the liver function, as the former was reversed to levels not statistically significant from control.

### 3.2. Effect of TQ on Tissue Oxidative, Nitrosative, and Inflammatory Markers in MTX-Treated Rats

GSH concentration, catalase activity, and MDA level were determined as markers of oxidative stress, whereas total nitrite/nitrate was assessed as an indicator of nitrosative stress and TNF-*α* as an inflammatory marker. MTX-treated group showed significant decrease in GSH concentration and catalase activity compared with untreated control in both kidney and liver (Tables 2 and 3, resp.). Concomitant treatment with MTX and TQ increased renal and hepatic GSH and catalase values to levels statistically higher than MTX-treated group. On the other hand, renal and hepatic MDA, NO, and TNF-*α* levels increased in MTX-treated group compared to control. This increase was reversed by combined treatment with MTX/TQ which showed significantly lower levels of these parameters compared to the group treated with MTX alone. Interestingly, GSH concentration, MDA level, and total nitrite/nitrate in kidney tissue but not the liver were improved in MTX/TQ group to levels not significantly different from control.

### 3.3. Effect of TQ on Renal and Hepatic iNOS Expression in MTX-Treated Rats

**Figure 1:**
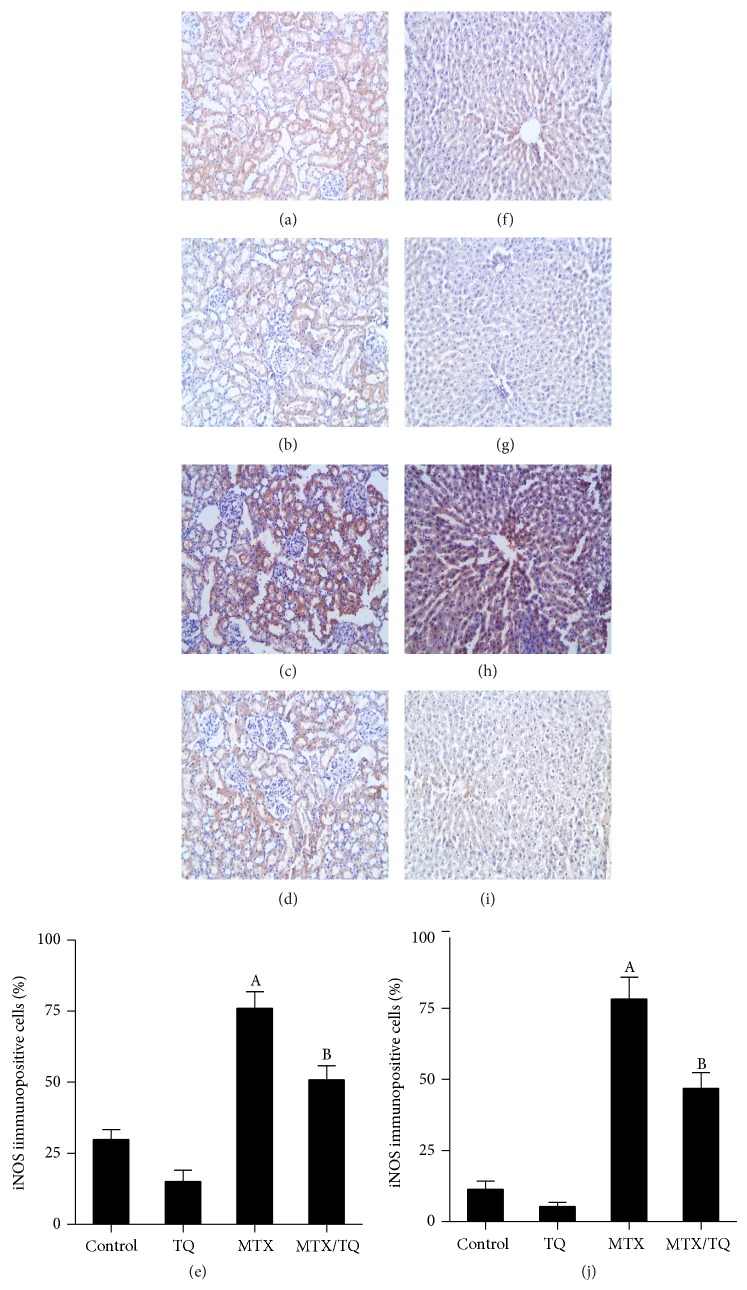
Effect of thymoquinone (TQ) on inducible nitric oxide synthase (iNOS) immunohistochemical staining of methotrexate- (MTX-) treated rat kidney and liver. Localization of iNOS immunoreactivity (×100) in the kidney (left panel) and liver (right panel) of (a and f) control group, (b and g) TQ-treated group, (c and h) MTX-treated group, and (d and i) concomitant MTX with TQ-treated group, respectively. (e and j) show semiquantitative analysis of iNOS immunohistochemical staining results in kidney and liver, respectively. Values are represented as means ± S.E.M. ^A^Significant difference compared with control, ^B^significant difference compared with MTX group. Significant difference is reported when *p* < 0.05.

Immunohistochemical staining of rat kidney and liver sections with iNOS (Figure 1) was performed to confirm nitrosative stress. ImageJ analysis was performed to calculate the degree of significance of protein expression, quantified by percent of immunopositive cells in kidney and liver tissue (Figures 1(e) and 1(j), resp.). Immunohistochemical staining of control rat kidney with iNOS revealed its expression in peritubular capillaries and tubular epithelial cells (Figure 1(a)). Comparable expression was observed in TQ-treated group (Figure 1(b)). On the other hand, MTX treatment caused significant increase in iNOS expression (Figure 1(c)), which was reversed in MTX/TQ group (Figure 1(d)). Immunohistochemical staining of control and TQ rat liver with iNOS revealed mild expression in the portal and sinusoidal vascular endothelia (Figures 1(f) and 1(g), resp.). MTX-treated group, however, showed significant increase in iNOS expression (Figure 1(h)), which was reversed in MTX/TQ group (Figure 1(i)).

### 3.4. Effect of TQ on Renal and Hepatic Histopathology in MTX-Treated Rats

**Figure 2:**
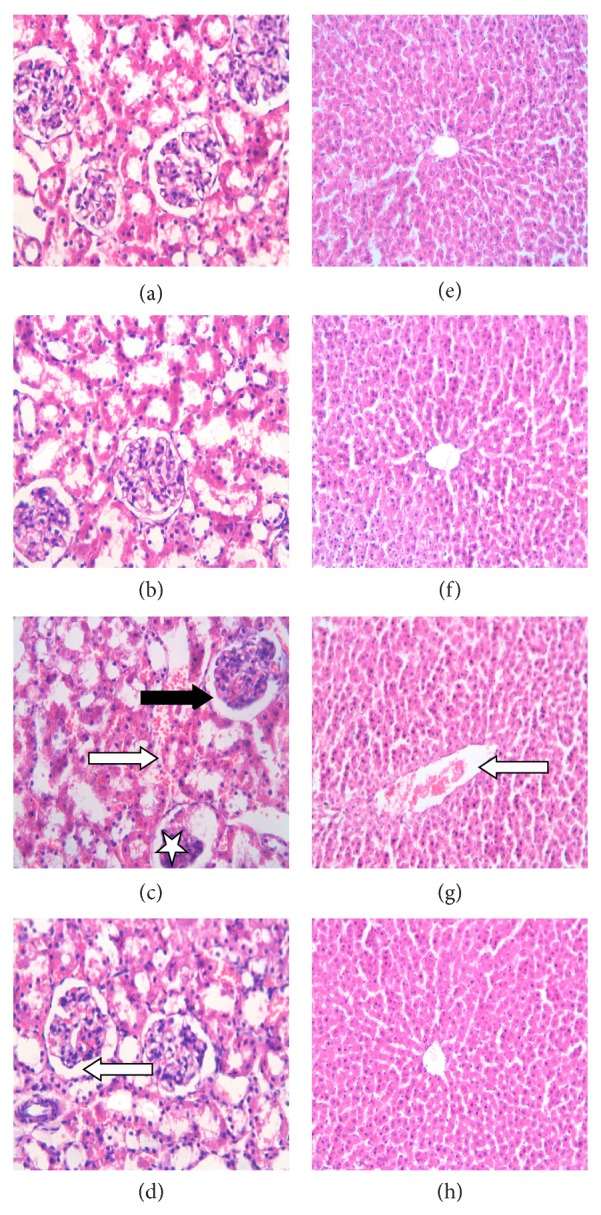
Effect of thymoquinone (TQ) on histology of the kidney and liver in methotrexate- (MTX-) induced toxicity in rats. Representative histological photomicrographs of hematoxylin and eosin-stained renal (left panel) and hepatic (right panel) sections (×40 and ×20, resp.) showing (a and b) control and TQ-treated groups, respectively, having normal structure of renal glomeruli and tubules, (c) MTX-treated group with glomerular atrophy (star), wide capsular space (black arrow), sloughing of epithelial cells of renal tubules, dilatation, and intratubular cellular casts (white arrow), and (d) MTX/TQ-treated group with mild dilatation of renal tubules (white arrow). Liver of both control and TQ-treated groups (e and f, resp.) has normal hepatic architecture, MTX-treated group (g) has dilated congested portal vein (white arrows), and MTX/TQ-treated group (h) shows normal hepatic architecture.

Kidney histopathological examination revealed that control and TQ groups had normal structure of glomeruli and renal tubules (Figure 2; left panel). MTX-treated group, on the other hand, presented with atrophied glomeruli having widened capsular spaces. In addition, most of the tubules were dilated and had intratubular cellular casts. Treatment with MTX/TQ improved renal histology, with only mild dilatation of renal tubules. Liver histopathological examination showed normal liver structure in both control and TQ-treated groups with normal lobular architecture, central vein, and radiating hepatic cords (Figure 2; right panel). To the contrary, treatment with MTX caused dilatation and congestion of portal vein. Combined treatment with MTX/TQ showed normal hepatic architecture.

### 3.5. Effect of TQ on Renal and Hepatic NF-*κ*B and COX-2 Expression in MTX-Treated Rats

**Figure 3:**
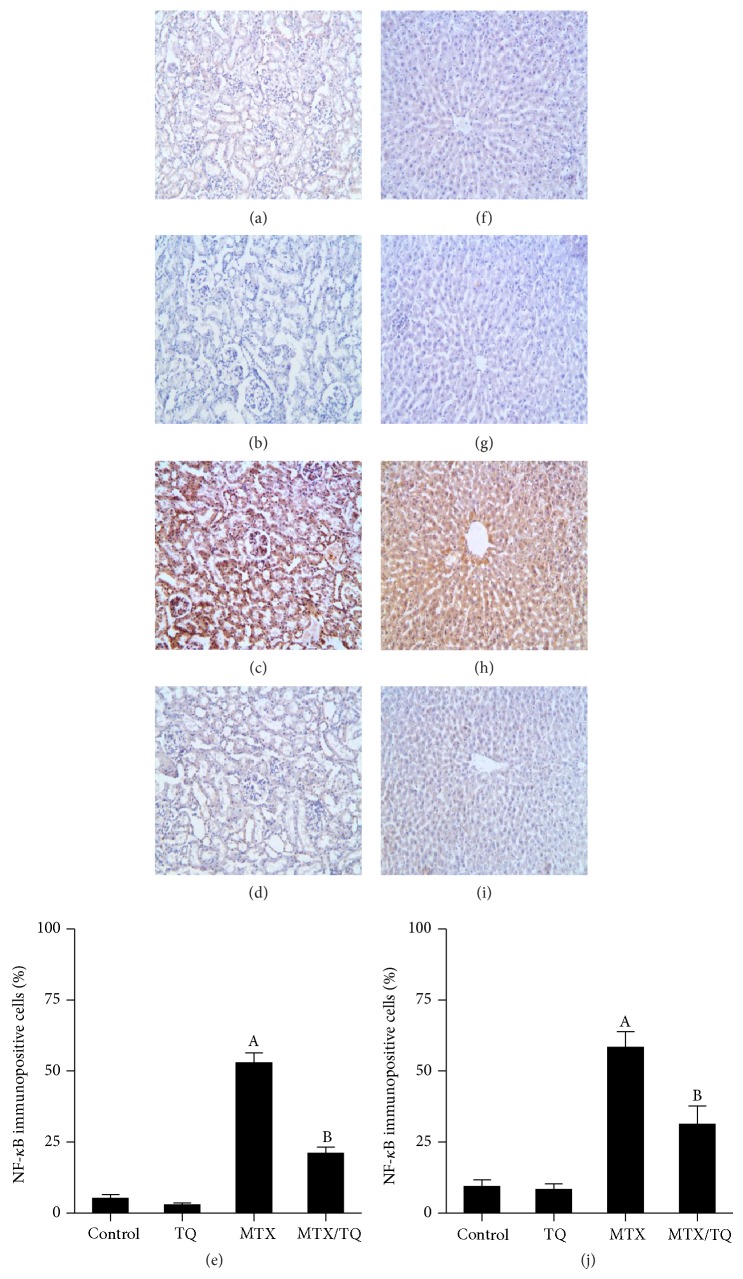
Effect of thymoquinone (TQ) on nuclear factor-*κ*B (NF-*κ*B) immunohistochemical staining of methotrexate- (MTX-) treated rat kidney and liver. Localization of NF-*κ*B immunoreactivity (×100) in the kidney (left panel) and liver (right panel) of (a and f) control group, (b and g) TQ-treated group, (c and h) MTX-treated group, and (d and i) concomitant MTX/TQ-treated group, respectively. MTX-treated group shows immunopositivity in the nuclei of renal tubular cells (c) and hepatocytes (h) which was markedly reduced in concomitant MTX/TQ-treated group (d and i). (e and j) show semiquantitative analysis of NF-*κ*B immunohistochemical staining results in kidney and liver, respectively. Values are represented as means ± S.E.M. ^A^Significant difference compared with control, ^B^significant difference compared with MTX group. Significant difference is reported when *p* < 0.05.

**Figure 4:**
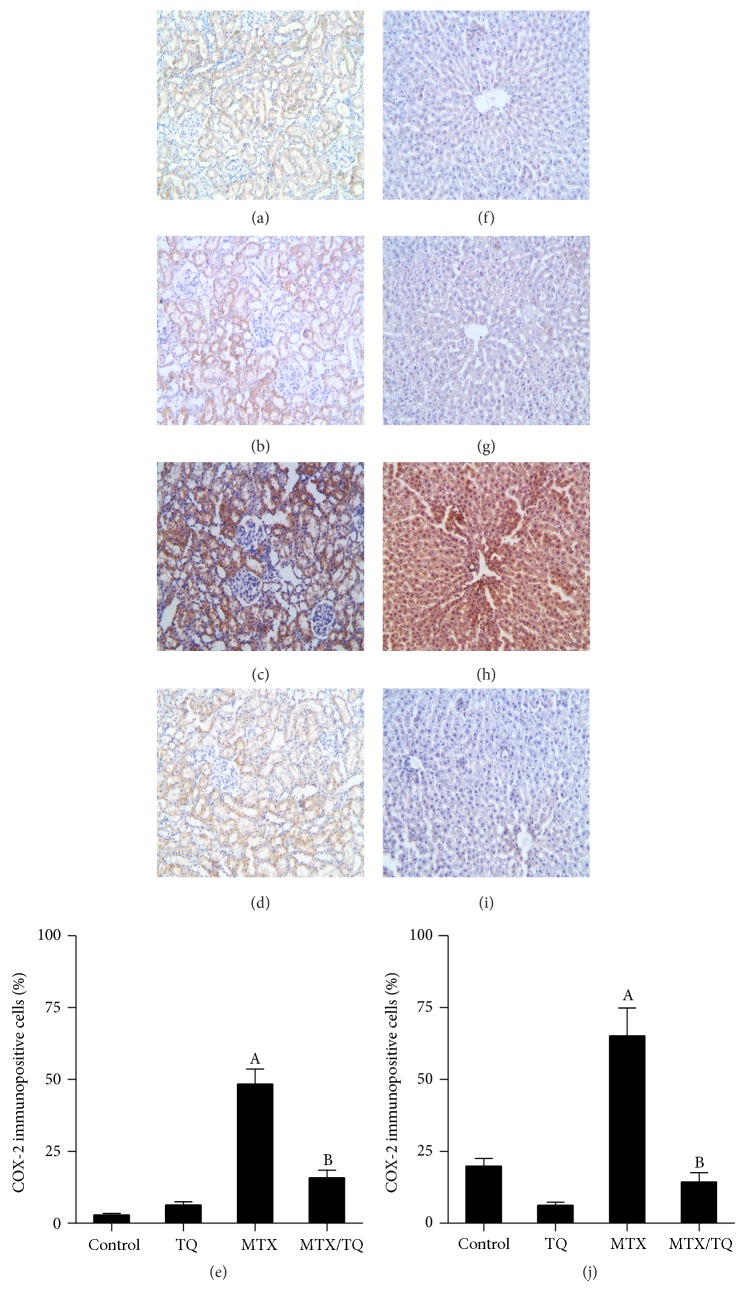
Effect of thymoquinone (TQ) on cyclooxygenase (COX)-2 immunohistochemical staining of methotrexate- (MTX-) treated rat kidney and liver. Localization of COX-2 immunoreactivity (×100) in the kidney (left panel) and liver (right panel) of (a and f) control group, (b and g) TQ-treated group, (c and h) MTX-treated group, and (d and i) concomitant MTX/TQ-treated group, respectively. (e and j) show semiquantitative analysis of COX-2 immunohistochemical staining results in kidney and liver, respectively. Values are represented as means ± S.E.M. ^A^Significant difference compared with control, ^B^significant difference compared with MTX group. Significant difference is reported when *p* < 0.05.

Immunostaining of rat kidney and liver with NF-*κ*B and COX-2 (Figures 3 and 4, resp.) was done to confirm inflammatory pathway involvement. Kidneys from control (Figure 3(a)) and TQ-treated (Figure 3(b)) groups showed mild cytoplasmic expression of NF-*κ*B in renal tubules, indicating that NF-*κ*B was present in its inactive form. In kidneys of rats treated with MTX alone, NF-*κ*B was observed to be highly expressed in renal nuclei (Figure 3(c)), indicating nuclear translocation, upregulation, and activation of NF-*κ*B. Combined TQ-treatment with MTX caused less staining of NF-*κ*B in renal nuclei (Figure 3(d)). In liver sections from control and TQ-treated groups, NF-*κ*B expression was mildly expressed in the cytoplasm of hepatocytes (Figures 3(f) and 3(g), resp.). Following MTX treatment, NF-*κ*B immunoreactivity was highly expressed in the nucleus (Figure 3(h)). A marked decrease in NF-*κ*B staining was observed in the nucleus of combined MTX and TQ therapy, with mild cytoplasmic staining (Figure 3(i)). For COX-2 immunostaining, mild staining was observed in kidneys of control and TQ-groups (Figures 4(a) and 4(b), resp.). MTX treatment caused significant increase in COX-2 expression in renal tubules (Figure 4(c)), whereas combined MTX/TQ treatment significantly decreased COX-2 expression (Figure 4(d)) compared to MTX alone. Similar findings were seen in COX-2 expression in the liver, where control and TQ groups showed minimal expression of COX-2 (Figures 4(f) and 4(g), resp.). MTX caused marked upregulation of COX-2 particularly in hepatocytes cytoplasm (Figure 4(h)). Coadministration of MTX/TQ significantly decreased COX-2 expression (Figure 4(i)) compared to MTX alone.

### 3.6. Effect of TQ on Renal and Hepatic Caspase 3 Expression in MTX-Treated Rats

**Figure 5:**
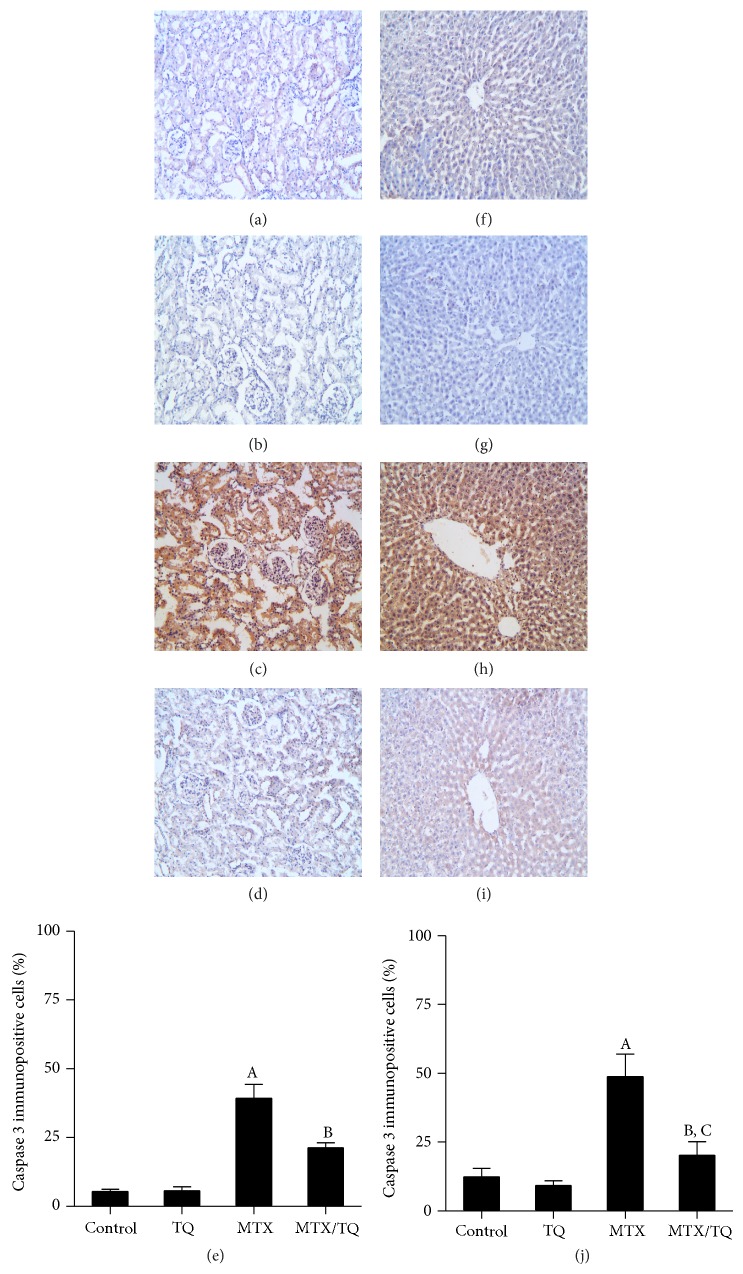
Effect of thymoquinone (TQ) on caspase 3 immunohistochemical staining of methotrexate- (MTX-) treated rat kidney and liver. Localization of caspase 3 immunoreactivity (×100) in the kidney (left panel) and liver (right panel) of (a and f) control group, (b and g) TQ-treated group, (c and h) MTX-treated group, and (d and i) concomitant MTX/TQ-treated group, respectively. (e and j) show semiquantitative analysis of caspase 3 immunohistochemical staining results in kidney and liver, respectively. Values are represented as means ± S.E.M. ^A^Significant difference compared with control, ^B^significant difference compared with MTX group, ^C^no significant difference compared to control. Significant difference is reported when *p* < 0.05.

Immunostaining of rat kidney and liver with caspase 3 (Figure 5) was performed as a marker of apoptosis. In the kidney of control and TQ groups, caspase 3 expression was minimal (Figures 5(a) and 5(b), resp.). In MTX-treated group (Figure 5(c)), however, caspase 3 was significantly upregulated especially in the epithelial lining of renal tubules. This expression was significantly reversed in MTX/TQ group (Figure 5(d)). In the liver, caspase 3 was minimally expressed in control and TQ group livers (Figures 5(f) and 5(g), resp.). To the contrary, caspase 3 expression was significantly increased in MTX-treated group (Figure 5(h)). On the other hand, combined MTX/TQ significantly decreased caspase 3 expression (Figure 5(i)) compared to MTX alone.

## 4. Discussion

MTX remains one of the most commonly used systemic therapeutic in treatment of cancer and autoimmune diseases. Several hypotheses have been suggested for the mechanisms underlying MTX toxicity, including the involvement of oxidative stress, inflammation, and apoptosis [2, 8, 11–14]. Here, we proved that one of the constituents of* Nigella sativa*, TQ, with well-documented powerful antioxidant properties [15], confers protection against MTX-induced hepatorenal damage. We also proved that the mechanisms of such protection included ameliorating MTX-induced oxidative and nitrosative stress, downregulation of TNF-*α*/NF-*κ*B/COX-2 inflammatory pathway, and inhibition of apoptosis.

In the present study, MTX caused deterioration of hepatic and renal functions, evident by alteration of BUN, creatinine, ALT, and AST, confirmed by the distorted histological picture of both organs, which was in agreement with previous studies [2, 14]. The mechanisms involved in MTX-induced hepatorenal damage were investigated and our results showed that MTX induced oxidative stress, evident by decreased GSH level and catalase activity with increased lipid peroxidation product, MDA, which was in concurrence with previous studies [2, 14]. Here, MTX also caused hepatorenal nitrosative stress, shown by increase in total nitrite/nitrate which is a representative of NO level, confirmed by upregulation of iNOS expression in both kidney and liver. MTX-induced increase of NO in the kidney [2, 16] and hepatocytes [12] has been previously reported. MTX was also reported to induce iNOS in the small intestine [17] and the brain [18]. However, to our best knowledge, no confirmation of MTX induction of iNOS expression in kidney or liver tissue has been suggested so far.

In the present study, we show that MTX increased hepatorenal tissue levels of TNF-*α* and activated, translocated, and increased the expression of NF-*κ*B, as well as the expression of COX-2. Previous studies have reported that MTX increased the levels of TNF-*α* and NF-*κ*B in kidney [8] and liver [12, 19]. On the other hand, COX-2 is an inducible enzyme that governs the transformation of arachidonic acid into prostaglandins as a part of inflammatory process. The promoter region of COX-2 gene has two motifs comprising the binding sites for NF-*κ*B [20], that, when bound, induces the expression of COX-2 in the kidney [21] and liver [22]. We recently demonstrated that MTX treatment markedly increased COX-2 expression in different degenerated cortical and medullary tubules [2]. Moreover, Mukherjee et al. [12] reported that MTX significantly increased COX-2 level in mice liver.

In the present study, MTX was shown to have proapoptotic effect via increasing the expression of caspase 3. MTX-induced increase of caspase 3 has recently been reported in liver [12] and in our study on kidney [8]. TQ has been reported to offer renal and/or hepatic protection in several experimental models, including diabetic nephropathy [23], gentamicin- [24] and vancomycin- [25] induced nephrotoxicity, cadmium- [26], acetaminophen- [27], tamoxifen- [28], cisplatin- [29], and aflatoxin- [30] induced hepatotoxicity, and hepatorenal damage associated with ischemia reperfusion [7] and hyperglycemia [31]. Nevertheless, few studies reported that TQ might confer protection against MTX toxicity itself [32].

In the present study, MTX/TQ group receiving 10 mg/kg TQ for 10 days and 20 mg/kg MTX given as a single dose at the end of day 3 of the experiment showed improved renal function as well as renal histology compared to MTX alone. Here, we also show for the first time that TQ ameliorates the decline in liver function induced by MTX, as it decreased ALT and AST as well as improving the histology of the liver compared to MTX alone. The mechanisms involved included reverting MTX-induced hepatorenal oxidative stress, as indicated by the increase in GSH level and catalase activity and the decrease in MDA. In addition, TQ reverted MTX-induced inflammatory signs, as it significantly decreased the level of TNF-*α*, NF-*κ*B, and COX-2, which is in line with the previously reported antioxidant/anti-inflammatory effects of TQ [33]. Furthermore, TQ reversed MTX-induced nitrosative stress in both kidney and liver as evident by decreasing NO tissue levels compared to MTX alone, which is in agreement with previous studies [34, 35]. Here, we show that TQ-induced decrease in NO level is due to downregulation of iNOS expression in kidney and liver. We also show that TQ had antiapoptotic effect that decreased the expression of the apoptotic marker, caspase 3 induced by MTX, which is in line with previous studies reporting TQ-mediated suppression of caspase 3 caused by endotoxemia in liver [36]. Interestingly, TQ is being currently investigated as a novel anticancer agent that promotes apoptosis by induction of expression of caspase 3 in a number of types of malignancies in vitro and in vivo [37]. The explanation of such discrepancy is that TQ has preferential apoptotic mechanistic effect that targets only tumor cells [38].

We have recently reviewed the interaction between oxidative, nitrosative, inflammatory, and apoptotic pathways [39], showing how complicated it is to determine if the relationship between these pathways is a cause or a consequence of one another. Still, we hypothesize that MTX initiates hepatorenal damage by induction of oxidative stress, which, in turn, activates TNF-*α*/NF-*κ*B/COX-2 inflammatory pathway, and such inflammatory process triggers automated cell death, apoptosis. TQ, as a powerful antioxidant, confers protection against MTX-induced toxicity via inhibiting the initiation of oxidative stress.

## 5. Conclusion

Prophylactic use of TQ succeeded in protecting against MTX-induced hepatorenal toxicity, through TQ-mediated antioxidant, antinitrosative, anti-inflammatory, and antiapoptotic mechanisms. Since TQ is now being tried as a possible anticancer agent, combined TQ hepatorenal protection proved in the present study and its antitumor activity draw the attention to TQ as a possible successful adjuvant in MTX chemotherapy.

## Figures and Tables

**Table 1:** Effect of thymoquinone (TQ) on change (Δ) of total body weight (wt), organ/total wt ratios, and kidney and liver function tests in methotrexate- (MTX-) induced toxicity.

	Control	TQ	MTX	MTX/TQ
Δ body wt (%)	100 ± 5	99 ± 4	88 ± 2^a^	98 ± 8^b,c^
Kidney/wt ratio	4.9 ± 0.4	5.03 ± 0.61	6.98 ± 1.19^a^	5.82 ± 0.96^b,c^
Liver/wt ratio	176.5 ± 16.3	185.3 ± 33.1	369.1 ± 34.8^a^	226.6 ± 26.4^b^
BUN (mg/dL)	4.4 ± 0.9	3.9 ± 1.1	8.1 ± 1.8^a^	5.9 ± 0.9^b,c^
Creatinine (mg/dL)	0.71 ± 0.38	0.81 ± 0.27	2.19 ± 0.48^a^	1.27 ± 0.72^b,c^
ALT (U/dL)	24.8 ± 6.3	29.8 ± 6.2	51.9 ± 4.1^a^	41.8 ± 6.3^b^
AST (U/dL)	129.3 ± 24.9	149.6 ± 12.2	199.3 ± 29.1^a^	162.9 ± 11.8^b^

Kidney/wt and liver/wt are ratios of weight of respective organ/total body weight *∗* 1000 ratio. BUN; blood urea nitrogen, ALT; alanine aminotransferase, and AST; aspartate aminotransferase. Values are representation of 8–11 observations as means ± SEM. Results are considered significantly different when *p* < 0.05.^ a^Significant difference compared to control, ^b^significant difference compared to MTX group, and ^c^no significant difference compared to control.

**Table 2:** Effect of thymoquinone (TQ) on reduced glutathione (GSH), catalase, malondialdehyde (MDA), nitric oxide (nitrite/nitrate; NO), and tumor necrosis factor- (TNF-) *α* levels in rat kidney homogenate exposed to methotrexate (MTX).

	Control	TQ	MTX	MTX/TQ
GSH (*μ*mol/g tissue)	12.9 ± 2.4	11.2 ± 3.3	5.1 ± 2.3^a^	9.5 ± 4.3^b,c^
Catalase (U/g tissue)	9.4 ± 0.9	9.9 ± 0.9	6.1 ± 0.9^a^	7.8 ± 0.5^b^
MDA (nmol/g tissue)	17.8 ± 8.4	21.3 ± 5.2	42.9 ± 11.4^a^	25.1 ± 6.3^b,c^
NO (nmol/0.1 g tissue)	85.4 ± 12.4	78.3 ± 14.6	156.8 ± 13.6^a^	100.1 ± 18.3^b,c^
TNF-*α* (ng/g tissue)	99.3 ± 16.1	103.8 ± 11.3	170.4 ± 14.6^a^	125.5 ± 12.8^b^

Values are represented as means ± SEM of 8–11 observations. ^a^Significant difference compared to control, ^b^significant difference compared to MTX, and ^c^no significant difference compared to control. Significant difference is reported when *p* < 0.05.

**Table 3:** Effect of thymoquinone (TQ) on reduced glutathione (GSH), catalase, malondialdehyde (MDA), nitric oxide (nitrite/nitrate; NO), and tumor necrosis factor- (TNF-) *α* levels in rat liver homogenate exposed to methotrexate (MTX).

	Control	TQ	MTX	MTX/TQ
GSH (*μ*mol/g tissue)	25.3 ± 6.3	27.4 ± 4.3	12.3 ± 3.3^a^	18.9 ± 5.3^b^
Catalase (U/g tissue)	13.2 ± 1.1	12.9 ± 0.9	5.2 ± 1.8^a^	9.1 ± 2.1^b^
MDA (nmol/g tissue)	34.1 ± 9.3	39.5 ± 11.4	62.9 ± 19.3^a^	52.4 ± 10.8^b^
NO (nmol/0.1 g tissue)	76.4 ± 12.1	79.3 ± 8.9	122.2 ± 14.1^a^	96.1 ± 14.2^b^
TNF-*α* (ng/g tissue)	49.2 ± 6.1	53.1 ± 10.3	129.7 ± 21.5^a^	81.7 ± 24.1^b^

Values are represented as means ± SEM of 8–11 observations. ^a^Significant difference compared to control, ^b^significant difference compared to MTX. Significant difference is reported when *p* < 0.05.
